# Hystology findings' correlation between the ossicular chain in the transoperative and cholesteatomas

**DOI:** 10.1016/S1808-8694(15)31169-1

**Published:** 2015-10-19

**Authors:** Cristina Dornelles, Letícia Petersen Schmidt Rosito, Luíse Meurer, Sady Selaimen da Costa, Andréia Argenta, Sabrina Lima Alves

**Affiliations:** 1 M.Sc. in Medical Sciences - Pediatrics, Doctorate student at Post-Graduation Course in Medical Sciences: Pediatrics - UFRGS, Biologist at the Brazilian Center for Otitis Media. Professor at IPA Post-Graduation Course in Speech Therapy. Invited Professor at the School of Medicine of UFRGS; 2 Ear, Nose and Throat Physician, M.Sc. Student in Medical Sciences: Surgery; 3 Doctor in Medical Sciences: Gastroenterology, Substitute Professor; 4 Doctor in Surgery. Associate Professor; 5 Scholar in Medicine; 6 Scholar in Medicine

**Keywords:** ossicular chain, cholesteatoma, inflammation, chronic otitis media, perimatrix

## Abstract

Chronic otitis media is hystopathologycaly defined as the presence of irreversible inflammatory tissue changes in the middle ear. Ossicular lesions represent the most prevalent change.

**Aim:**

to correlate the degree of ossicular chain changes seen during surgery with the inflammatory histological degree and the thickness of the cholesteatoma perimatrix.

**Study design:**

Cross-sectional study.

**Methods:**

Seventy-one descriptions of surgeries done in patients submitted to tympanomastoydectomy were reviewed. Cholesteatoma were collected and fixed in 10% formaldehyde. Two slides were made for each cholesteatoma, one stained with HE and another with picrossirius. Images were obtained from light microscopy and digitally processed and “blindly” analyzed using Image Pro-Plus Software. For statistical analysis we used Spearman's coefficient. Differences were considered statistically significant if P≤0.05.

**Results:**

the ossicular chain was involved in 65 cases. The incus was the most frequently affected bone, followed by the stapes and the malleus. When the Spearman's coefficient was employed considering ossicular chain change degree with patient's age by the time of surgery, perimatrix thickness and histological degree of inflammation, correlations were not established.

**Conclusion:**

Our findings indicate that ossicular chain changes are practically universal when a cholesteatoma is present. We didn't find correlations related with bone erosion and cholesteatoma's histological findings.

## INTRODUCTION

The presence of cholesteatoma at the ear cleft of patients with chronic otitis media, undoubtedly causes more morbimortality due to the great bone erosion power of these epithelial accumulations.^1^,^2^

They usually reach the ossicular chain and in a lesser extent the skull bones. Even the most rigid bone in the human body, the optic capsule, is affected, showing its strong destroying action on the bone tissue. Partial or total destruction of ossicles is seen in approximately 80% of patients with cholesteatoma, whereas in chronic otitis media without cholesteatoma, ossicular chain erosion can be seen in approximately 20% of the cases^3^. The mechanisms leading to this increase in bone degradation in the presence of cholesteatoma are still unclear.^4^ According to Swartz^4^, ossicular destruction is certainly the most common problem among cholesteatoma complications and the type of destruction depends on its origin and the way it spreads itself. According to his data, ossicular chain is intact in only 26% of attical cholesteatomas. The most affected region is the long process of the incus followed by the incus body and the malleus head. On the other hand, pars tensa cholesteatomas show an erosion power of 90%.

Bone absorption is stimulated by several factors, including inflammation, local pressure and specific cytokeratins^5^. The enzymatic concept in which epithelial origin enzymes are considered to be responsible for bone destruction was defined by Abramson^6^, who showed the presence of collagenases and hydrolysis in cholesteatoma. This hypothesis was later confirmed by Thompsen^7^. Ferlito et al.^8^ suggested that the destructive property of cholesteatomas, bone erosion, is caused by the collagenases produced by the components of squamous and fibrous epithelial tissues. The hypothesis of bone absorption by biochemical action exclusively done by collagenolitic enzimes was later incorporated by other agents, such as tumor necrosis factor (TNF), interleukins (IL-1a) and prostaglandins (PGE2)^9^, ^10^, ^11^.

By the histologic analysis of cholesteatomas, Dornelles^12^ found an inverse correlation between the perimatrix size measured in micrometers and the age of patients at the day of surgery and that the degree of the perimatrix inflammation was strongly correlated with the perimatrix thickness. However, in this paper we did not evaluate the relationship between the degree of perimatrix inflammation and the ossicular involvement.

This piece of work wants to correlate the degree of the ossicular chain involvement seen at the trans-operative stage with the age of patients at the day of surgery, the histological degree of inflammation and the perimatrix thickness of acquired cholesteatomas.

## METHODOLOGY

The Research and Post-Graduation Group of this institution approved this piece of work on its ethical and methodological aspects with the number 03-271 in 2003. Due to its methodology it is classified as a comparative and contemporary cross study.

We reviewed the descriptions of trans-operative findings of 71 patients with chronic otitis media with cholesteatoma (COMC), followed up at the Chronic Otitis Media outpatient clinic, submitted to tympanomastoidectomy between March 2003 and June 2006.

Surgical descriptions were written by the resident physician who was the first assistant at the surgeries, with data from patients, pathology, conduct and specially the inspection of the ossicular chain which is thoroughly detailed.

In order to quantify the degree of ossicular erosion the following scale was prepared adding up the score according to the items: 0 - if the ossicular chain is intact; 1 - for discontinuity of the chain; 1 - for each eroded ossicle; 2 - absence of ossicle; 3 - eroded footplate. This score was cumulative and in order to obtain the score of the patient every alteration found was added up, having as a result a scale with values between 0 and 10.

**Figure 1:**
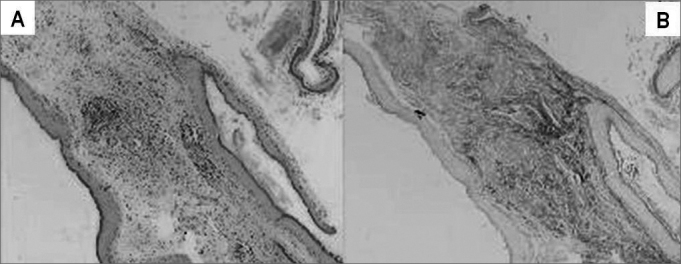
Same sample of stained cholesteatoma in HE (A) and Picrosirius (Sirius Red) (B).

Cholesteatomas were collected by the otologist surgeon, immediately fixed in formol at 10%, processed by regular histological techniques and embedded in paraffin. For the morphological analysis of each sample two slides were prepared. The slides were stained in Hematoxiline-Eosine (HE) and Picrossirius (Sirius Red) and analyzed in an optical microscope (Figure 1). Please note that in Figure 1 the contrast obtained in the second staining is much greater, since collagen fibers on them are stained in burgandy, which makes easier the differentiation of the perimatrix thickness to be measured. The reading of the material was “blind” and controlled by the researcher. The following constituents were observed: perimatrix thickness and inflammation.

For the histological degree of inflammation, an ordinal variable was created with values from zero to three, where 0 = absent, 1= discreet, 2 = moderate and 3= accentuated, characterized by the intensity of perimatrix permeation by lymphocytes, neutrophils, plasmocites and macrophages^13^.

**Figure 2:**
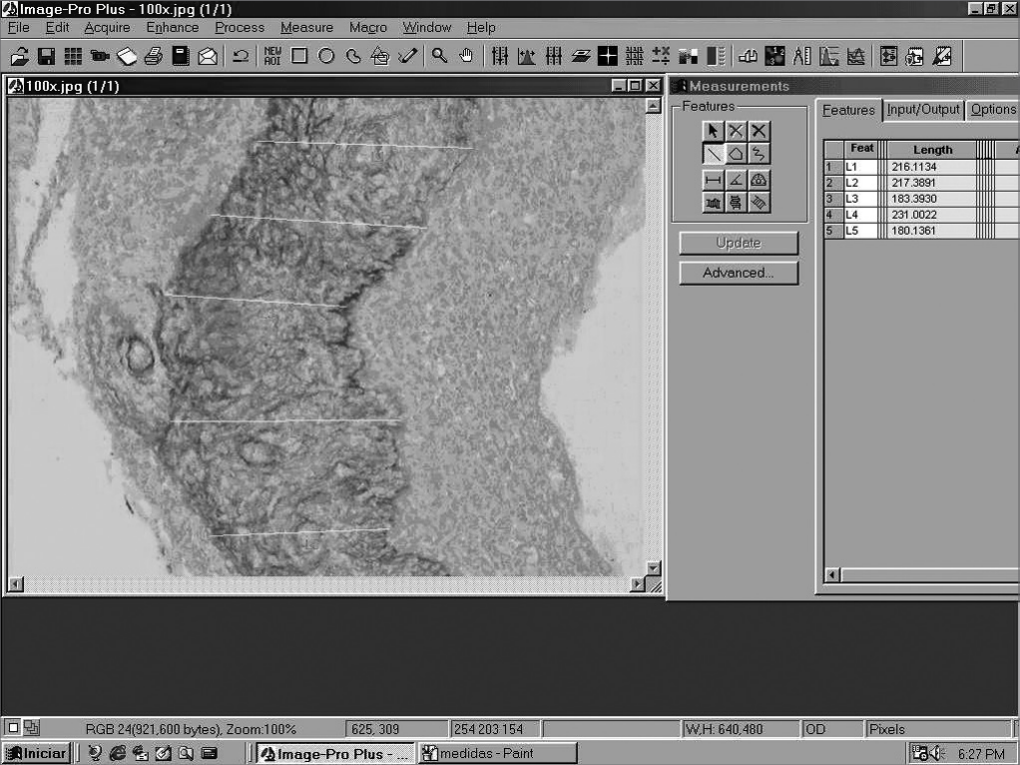
Screen image of ImagePro Plus Media Cybernetics software.

Perimatrix thickness was obtained through the analysis of computarized images using ImagePro Plus Media Cybernetics software (Figure 2). For each sample we took 20 perimatrix thickness measurements and they were summarized by the average. This was the parameter used to test the correlation of perimatrix thickness with the ossicular erosion degree.

Patients were classified into two groups according to their age at the day of surgery, as follows: Pediatrics - patients no older than 18 years old and Adults - patients older than 19 years old.

For the statistical analysis, we used Spearman's coefficient in the analysis of the correlation of ossicular erosion degree with the histological degree of inflammation, with perimatrix thickness and age of patient at the day of surgery. For descriptive data we use frequency tables and the comparison between the groups was done through a Student's t- test, using SPSS 10.0 software for Windows. P values below 0.05 were considered as statistically significant.

## RESULTS

### Epidemiological Data

The sample had 71 patients with ± standard deviation of 26±18 years old. In this group we had 38 patients being 18 years old or younger with ± standard deviation equal to 11±4 years old. There were 33 patients older than 18 years old, with ± standard deviation equal to 39±15 years old.

### Histological findings

Perimatrix appears as an inflammatory network surrounding the cholesteatoma with variable thickness both intra and inter-patients. The total group showed an average of 80 micrometers with interquartile interval of 37 to 232, with the minimum value zero and the maximum value 1.926.

When we analyzed inflammation degree at the perimatrix with the optical microscope, 60% of the samples were classified as moderate and accentuated.

### Trans-operative Data and Correlations

Ossicular chain was somehow compromised in 65 (92%) of the 71 patients reviewed (Table 1).

**Table 1:** State of ossicular chain in pediatric and adult groups.

State of the chain	Pediatric	Adult	Total
Complete	2	4	6
One eroded ossicle	5	5	10
Two eroded ossicles	10	3	13
Three eroded ossicles	7	5	12
One eroded ossicle and one absent ossicle	6	4	10
One eroded ossicle or two absent ossicles	1	3	4
Two eroded ossicles or one absent	2	6	8
Two eroded ossicles and stape footplate absent	3	1	4
Absence of ossicular chain	2	2	4
Total	38	33	71

When we analyzed each ossicle separately, the incus was the most frequently affected, which was absent in 14.2% of procedures and with erosion of its long process in 47.6%, followed by the stapes with erosion of its supra-structure in 32.3% and by malleus which was absent in 3.8% of the total number of ears.

**Figure 3:**
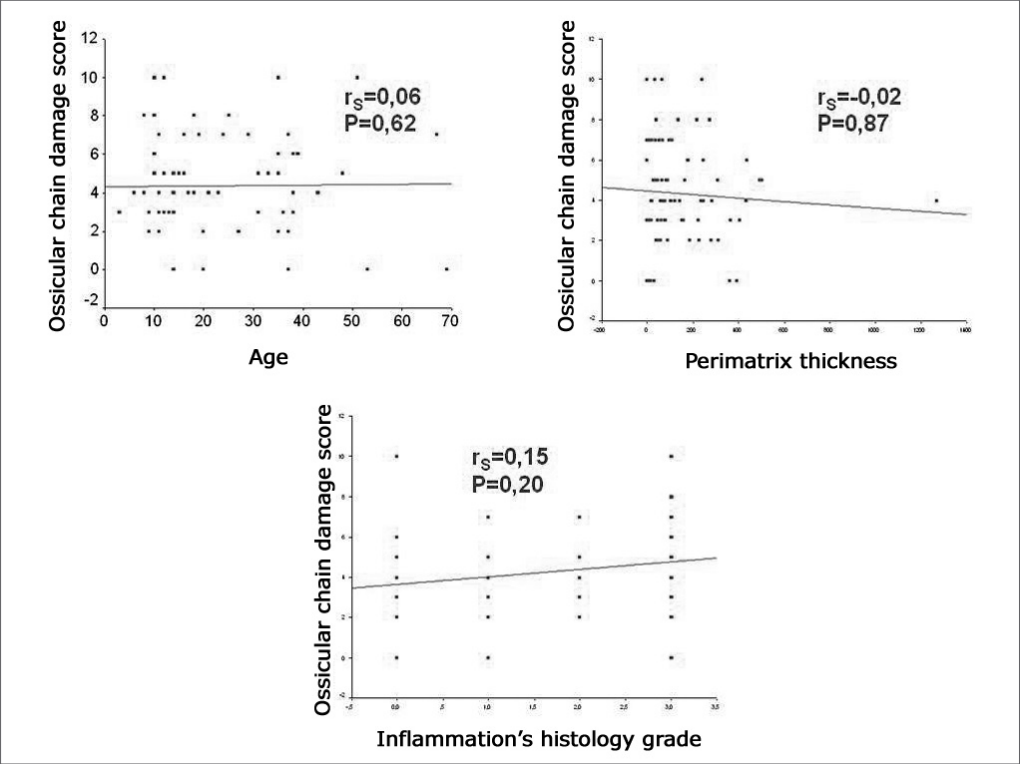
Scatter Plot between the degree of compromised ossicular chain and the age of patients at the day of surgery, perimatrix thickness and histological degree of inflammation.

The degree of compromise of the ossicular chain showed an average of 4.35 ± 2.52 in the sample, with 4.26 ± 2.37 in pediatric patients and 4.45 ± 2.73 in the adult group. When comparing the degree of involvement of the ossicular chain between the age groups through the Student's t- test, we did not find any statistically significant difference (P=0.753). When we applied Spearman's coefficient between the degree of involvement of the ossicular chain and the age of the patient at the day of surgery, perimatrix thickness and the histological degree of inflammation, we did not find any correlation (Figure 3).

## DISCUSSION

Ossicular alterations take part in the concept of chronic otitis media, that is to say, irreversible inflammatory tissue damages. This type of involvement has a clear clinical repercussion since it affects the basis of sound air conduction to the inner ear, having as an unavoidable result a air conduction dysacusia of varied intensity. It is believed that the type of alteration caused by the chronic inflammatory process in the ossicular chain follows a pattern that tends to be continuous and more or less repetitive. This means that the ossicular structure is kept in a fragile balance between its own resistance and the destructive mechanisms of chronic inflammation.

Sadé and Fuchs^14^ compared ossicular erosion findings in adults with those found in children. Destruction percentage in stapes and malleus was similar in both groups; however, the incus shows a significantly higher destruction in adults. In this study we did not find any differences in the score of both ossicular damage and ossicles separately between pediatric and adult groups.

In a previous study of our groups^15^, we did a description study of the middle ear findings at the transoperative stage of 55 patients with chronic otitis media, followed up at the Chronic Otitis Media outpatient clinic. In this group of patients 49% had the diagnosis of Chronic Otitis Media with Cholesteatoma (COMC). In the sample as a whole, there was some involvement of ossicular chain in 66%, in COMC this rate was 96% and in Chronic Otitis Media without Cholesteatoma was reduced to 37%. The presence of cholesteatoma was associated with the existence of two or more ossicles affected, as well as with the higher prevalence of absence or erosion of the ossicles. These findings indicate that most patients with COM, who underwent a surgical intervention has some impact in the ossicular chain and that the frequency and extension of this involvement were much more related to the presence of cholesteatoma.

In our study, the incus was the most affected ossicle by pathology, followed by the stapes and by the malleus respectively. The fact the incus is the most affected ossicle is maybe due to its incudal mass, to its prominent bone marrow and mainly, to the exposure and fragility of the long apophysis and its lenticular process. These factors acting synergically would make this ossicle more vulnerable to extrinsic aggressions and to osteomyelitis processes. These findings confirm those by Tos16, who reviewed ossicular pathology in 1.150 ears with chronic otitis media and indicated the incus, stapes and malleus sequence as the most frequently affected by the inflammatory process.

Today it is believed that ossicular defects are caused by active processes of bone resorption and not by ossicular necrosis. This theory presupposes the presence and participation of living cells at the demineralization, erosion and bone destruction mechanisms^17^. A simply necrotic bone can remain in situ for several years without suffering any resorption process. This possibility is well illustrated by the techniques of ossicular chain reconstruction with implants of homologous ossicles. In these situations, the ossicles are kept intact at the long run, allowing the propagation of sound stimulus through the middle ear.

Bone resorption mechanism in chronic otitis media is not completely understood. Ruedi^18^ and Tumarkin^19^ suggested that bone resorption would be due to the pressure placed by cholesteatomas on the ossicular surface. Thompsen et al.^20^ and Sadé & Berco^21^ noticed that eroded ossicles were unvariably surrounded by an inflammatory reaction and they suggested that the inflammation was the cause of ossicular resorption. It has been proved that the granulation tissue adjacent to the ossicles is able to produce a variety of enzymes and mediators that accelerate ossicular resorption; they include lysosomic enzimes^22^, collagenases^23^ and prostaglandins^24^. However, the dominating cell in bone resorption process still has controversies. Whereas some studies reveal the presence of osteoclasts in the areas of bone destruction^24^, others indicate mononuclear cells as being responsible for the situation^25^. According to some authors, the persistent inflammation on chronic otitis media with cholesteatoma would cause a constant healing process at the cholesteatoma perimatrix, with the consequent increase of cytokine levels. Among other factors, they could be responsible for cholesteatoma growth and for the bone destruction caused by it^26^.

In a previous study^12^, our group found several indications that the degree of histological inflammation would be strongly correlated with the perimatrix thickness of cholesteatomas. According to Mayot et al.^27^, in the case of chronic otitis media, the immune system defenses coming from the middle ear mucus would be recruited and would contribute to the pathogenia of the cholesteatoma. Therefore, perimatrix inflammation could contribute to the aberrant behavior of cholesteatoma^28^. Some authors believe that the granulation inflammatory tissue (perimatrix) needs bone erosion, which is a feature of the chronic otitis media with cholesteatoma.^4^,^29^,^30^

We could draw an analogy between the perimatrix and a “battlefield”, where there is a fight for the middle ear territory. On the one hand, on the attack we have the cholesteatoma; on the other hand, the adjacent tissues of the tympanic box mucus. With the expansion of cholesteatoma, the inflammatory reaction would increase and therefore, it would produce more elements of the inflammatory cascade.

This was the perspective we used to do this study, in order to verify the influence of perimatrix and its inflammatory state during the ossicular erosion process; different from what our hypothesis suggested, we did not find any correlation between the histological and trans-operative findings in our study cases.

Bone erosion resulting in chronic otitis media with cholesteatoma can be classified based on two hypothesis: biophysical and biochemical. Biophysical action basis would be the pressure done by the cholesteatoma on the ossicular chain and on the middle ear walls. On the other hand, the biochemical hypothesis is based on the destructive action of collagenases and inflammatory products.

With the methodology used in this study, we managed to do an indirect quantification of the biochemical action, although until now biophysical action does not have any methodology for measurement, therefore the fact that we did not find any correlation between the perimatrix thickness (indirect measurement of collagenases) and the histological degree of inflammation (indirect measurement of inflammatory products) may not mean that it does not exist, but it could be influenced by the pressure factor of the cholesteatoma on the ossicular chain, that would work as a confusing factor on this analysis.

Another fact that may influence the results of this study is the long waiting time for surgery, since our patients came from the public health system. This means they had to face a waiting period of two to five years until surgery once the diagnosis was done. This long waiting time could be responsible for the advanced stage of ossicular erosion found on this sample.

Thus, cholesteatomas biology has to be better understood in order to be able to elucidate the real mechanisms that destroy the ossicular system. Studies on this issue are being done by our group through the immunohistochemical analysis of collagenases and angiogenesis.

## CONCLUSIONS

Most patients with chronic otitis media with cholesteatomas who underwent surgery have some impact on the ossicular chain. It can be seen that ossicular destruction followed a trend of steps, beginning at the incus, then the stapes until reaching the malleus. All these findings corroborate those found in the literature.

We did not find any correlation between the ossicular erosion degree and the histological findings.

## ACKNOWLEDGMENTS

We thank the Otolaryngology and Pathology Services at our institution for allowing us to use their infrastructure for the development of this project.

We also thank the Research and Post-Graduation Group at our institution for their technical support.

We thank the Research and Events Incentive Fund at our institution for the financial support.
